# Toward Perceptive Soft Robots: Progress and Challenges

**DOI:** 10.1002/advs.201800541

**Published:** 2018-07-13

**Authors:** Hongbo Wang, Massimo Totaro, Lucia Beccai

**Affiliations:** ^1^ Center for Micro‐BioRobotics Istituto Italiano di Tecnologia Viale Rinaldo Piaggio 34 56025 Pontedera (Pisa) Italy

**Keywords:** proprioception, robotic sensing, soft robotics, soft sensors, tactile sensing

## Abstract

In the past few years, soft robotics has rapidly become an emerging research topic, opening new possibilities for addressing real‐world tasks. Perception can enable robots to effectively explore the unknown world, and interact safely with humans and the environment. Among all extero‐ and proprioception modalities, the detection of mechanical cues is vital, as with living beings. A variety of soft sensing technologies are available today, but there is still a gap to effectively utilize them in soft robots for practical applications. Here, the developments in soft robots with mechanical sensing are summarized to provide a comprehensive understanding of the state of the art in this field. Promising sensing technologies for mechanically perceptive soft robots are described, categorized, and their pros and cons are discussed. Strategies for designing soft sensors and criteria to evaluate their performance are outlined from the perspective of soft robotic applications. Challenges and trends in developing multimodal sensors, stretchable conductive materials and electronic interfaces, modeling techniques, and data interpretation for soft robotic sensing are highlighted. The knowledge gap and promising solutions toward perceptive soft robots are discussed and analyzed to provide a perspective in this field.

## 1 Introduction

In the last decade, soft robotics has been rising up as an emerging research topic,^1^ with some remarkable achievements such as: universal jamming gripper,^2^ multigait soft robot,^3^ worm robot,^4^ octopus robot,^5^ fully integrated soft octobot,^6^ growth robot,^7^ soft multilocomotion microrobot,^8^ and so on. Building on intrinsically soft materials or compliant mechanisms, soft robots are rapidly opening new possibilities for typical robotic tasks (e.g., grasping, dexterous manipulation, and locomotion), and also adding new robotic abilities that were unthinkable before,^9^ like morphing and self‐healing. All this is introducing new ways of supporting or merging with humans. Unlike their rigid counterparts, soft robots can actively and passively change their shape for safe, robust, and effective interactions by simple control methods.

Obtaining autonomy and going beyond open‐loop control requires integration of multimodal sensors into these soft‐bodied systems to provide sensory feedback. In particular, mechanosensing^10^ plays a vital role among many perception modalities. To perform their tasks as smoothly as biological systems, soft robots must perceive their own shape, namely proprioception, and be able to feel external stimuli, namely exteroception. In biological systems, different receptors are employed to convey information on a multitude of parameters, like pressure, strain, and many others (e.g., temperature, light, sound, pain, and even chemicals). However, the hallmark of perception is the detection of both external and internal mechanical cues, which enables living organisms to thrive in the world.^11^ Therefore, in this work, we focus on mechanical sensing (proprioception and tactile sensing) for soft robots.

Proprioception for soft robots is much more difficult than their rigid counterparts, because they have almost infinite degrees of freedom (DOFs) and can be deformed by both internal driving and external loads. First, it is difficult to accurately predict the response of a soft robot due to certain driving condition based on modeling because of the complex behaviors (nonlinearity, hysteresis, viscoelastic effect, large strain, or deformation) of these hyperelastic materials used,^12^ or compliant structures.[[qv: 4c]] A tiny mismatch between the model and the physical system could lead to a completely different result.^13^ Second, soft robots' shape/status/position can be passively changed by unknown external loads. Therefore, a soft robot cannot perform a task accurately with open‐loop control even in a well‐constructed environment.

Soft robots are inherently safe, and are flexible to a range of tasks (e.g., universal gripper), benefiting from passive adaptation of their elastic body to the objects they interact with. However, tactile sensing is still crucial for controlling robots in real‐world scenarios. Since soft robots can be deformed by external mechanical cues (e.g., contact and interaction forces), they could fail at some cases (e.g., locomotion over rough terrain, or in crowded scenarios) unless tactile sensing feedback is provided. Moreover, tactile sensing is essential for skilled tasks (e.g., sorting of objects based on surface texture, dexterous manipulations, etc.), effective exploration of the unknown world, and interaction with humans and the environment.

**Figure 1:**
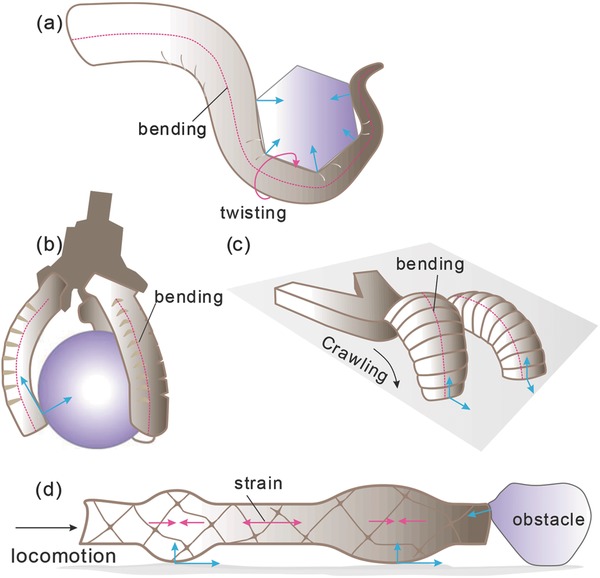
Schematics of some common type of soft robotic systems capable of: i) grasping and manipulation, like a) a continuum arm, and b) a three‐finger gripper; and ii) locomotion and exploration, like c) a crawling robot, and d) a worm‐like robot. Some of the mechanical parameters that could be provided as sensory feedback are exemplified (red lines and arrows for proprioception, blue arrows for tactile sensing).

Several types of soft robots (**Figure** **1**) have been developed to perform tasks like grasping, manipulation, locomotion, and exploration. Their capabilities could be significantly enhanced by enabling both proprioception and tactile sensing, including strain, pressure, bending, twisting, etc. As illustrated in Figure 1d, using a worm robot as an example, compressive and tensile strain distribution can be used for closed‐loop control of locomotion; contact pressure and shear force with the ground can help the robot to adjust its shape over rough terrain; and tactile sensing at its head can provide useful feedback to detect and avoid obstacles.

Perception is essential toward autonomous and intelligent soft robots. There are many challenges for implementing mechanically perceptive soft robots, a major one is that there is no clear distinction between proprioception and tactile sensing when designing a sensing system for soft robots. Sensing has been investigated since the birth of soft robots,^14^ sustainable progress has been achieved, benefiting from advances in soft materials and structures, flexible and stretchable sensors, fabrication techniques, and flexible electronics. Nevertheless, soft robotics sensing is still at its infancy and relatively underdeveloped compared to actuation and stiffening in soft robotics. Since 2011, quite a few review papers on soft robotics have been published,[[qv: 1b,9,15]] focusing on one or a few of these aspects: design, fabrication, simulation, stiffening,[[qv: 15b]] or control.^16^ Some of them have briefly discussed the sensing aspect,[[qv: 15d]] but there is no comprehensive report on this specific topic yet. Similarly, there are lots of review papers on tactile sensors for conventional robotics^17^ and biomedical applications,^18^ and many other reviews on tactile sensing technology.^19^ Recently, Li et al.^20^ published a short review which summarized some flexible and stretchable sensors that have been integrated into fluidic elastomer‐actuated soft robots.

In this paper, we report developments in mechanical sensing for soft robots and the enabling technologies, providing a comprehensive discussion and analysis of the real knowledge gap toward effective mechanical perception systems for soft robots, to identify key barriers and to provide a perspective in this field.

## 2 Progress in Soft Robotics Sensing

To the authors' knowledge, research on integrating flexible and stretchable sensors into soft robots can track back to 2007 (excluding fixed soft parts, e.g., artificial fingertips^21^), and it has started to grow at a steady pace from 2014. Among all those developments in soft robotics sensing, most of them are focusing on proprioception only, very few have attempted to achieve both proprioception and tactile sensing at the same time. Furthermore, some researchers have investigated sensor configuration methods^22^ and shape reconstruction algorithms^23^ for certain type of soft robots.

### 2.1 Proprioception

**Figure 2:**
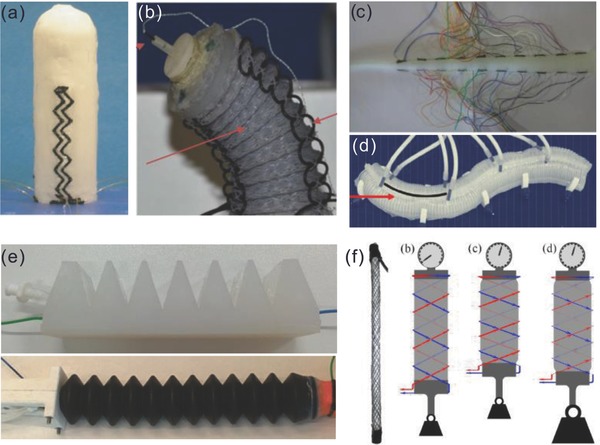
Examples of soft robotics proprioception. a) A soft bending actuator with integrated piezoresistive curvature sensor. Reproduced with permission.[[qv: 14b]] Copyright 2008, Elsevier. b) An omnidirectional actuator with integrated optical sensor for bending direction and angle measurement. Reproduced with permission.^35^ Copyright 2015, IOP Publishing. c) A soft continuum robot with integrated resistive sensor for shape reconstruction. Reproduced with permission.[[qv: 14c]] Copyright 2012, IEEE. d) A snake‐like soft robot with integrated magnetic sensor for bending angle monitoring. Reproduced with permission.[[qv: 42b]] Copyright 2015, Elsevier. e) Proprioceptive soft bending and elongation actuators driven by ionic liquid. Reproduced under the terms of the CC‐BY license.^43^ Copyright 2018, the Authors, published by Mary Ann Liebert. f) Self‐sensing McKibben muscles using the “smart braid” approach. Reproduced with permission.[[qv: 38a]] Copyright 2014, IEEE.

To date, investigations on soft robotics proprioception have covered pneumatic bending and elongation actuators and omnidirectional actuators, McKibben muscles, bionic and human fingers, soft continuum robots, and so on. Pneumatic actuators have attracted lots of interests since the innovations of PneuNet^24^ and fiber‐reinforced actuators;^25^ they are the basis on which many kinds of soft robots,^26^ prosthetic hands,^27^ and wearable systems^28^ can be built. Several stretchable strain sensors have been integrated into soft bending actuators to measure the bending angle (**Figure** **2**a), e.g., textile electrode–based capacitive sensor,^29^ liquid metal–based resistive sensor,^26^, ^30^ nanocomposite‐based piezoresistive sensor,[[qv: 14b,31]] engraved optical fiber sensor,^28^ and waveguide optical system.^27^ Pneumatic omnidirectional actuators usually have a cylindrical shape with three air chambers that are controlled by air pressure, which can bend in any direction. They have great potential in minimal invasive surgery (MIS) applications,^32^ where accurate position control is essential . Therefore, multiple strain sensors have been integrated into omnidirectional actuators to monitor both bending angle and direction (Figure 2b), e.g., ionic liquid–based resistive sensor,^33^ conductive yarn–based resistive sensor,^34^ optical fiber sensor.^35^ McKibben muscles were invented for orthotics in the 1950s, they have been well investigated and developed for many applications. Recently, the integration of sensors into McKibben muscles was addressed to measure the contraction length or circumference in real time by using several methods, like a piezoresistive ring,^36^ conductive fibers and yarns,^37^ and the “smart braid.”^38^ These case studies cited above focus on the system integration of different types of stretchable strain sensors into soft robots and actuators, and the validation of the sensing functionalities in these systems.

A variety of soft continuum robots^5^, ^39^ have been developed and explored due to their incredible capabilities for manipulation and motion, benefiting from their infinite DOFs and deformable body which make it difficult to track their shape. In 2012, Cianchetti et al.[[qv: 14c]] developed a sensing system to reconstruct the spatial configuration (in 2D) of a soft continuum robot by integrating ten pairs of textile‐based resistive sensors on the outer surface of the robotic arm (Figure 2c). In 2016, Wang et al.^40^ have developed a 3D shape reconstruction algorithm for a cable‐driven soft continuum arm by integrating 20 fiber Bragg gratings (FBGs) on four fibers inside the soft arm. A few other researchers have investigated polyvinylidene fluoride (PVDF) deflection sensors^41^ for shape reconstruction of a soft continuum arm, Hall‐effect sensors^42^ for monitoring the shape of a snake‐like robot (Figure 2d). To date, the reconstruction accuracy is still poor (particularly for large deformation). Furthermore, twisting and elongation in soft continuum robots are neglected in these studies because of their limited sensing capability. More sensing nodes, better sensor configuration, new modeling and reconstruction algorithms are needed to fully solve this problem.

Among all these work in proprioception, a few should be acknowledged due to the transformative nature of these concepts. In 2017, Helps and Rossiter^43^ developed ionic liquid–driven soft bending and elongation actuators, which are capable of self‐proprioception by monitoring the resistance variation of the driven fluid (Figure 2e). The temperature dependence of the ionic liquid sensor has been investigated too. Potentially, this type of sensor can be adapted for tactile sensing as well, similar to BioTac finger system^44^ (SynTouch Inc., USA). The application of this method is limited to fluid‐driven actuators, and cannot be used in the lightweight pneumatic actuators and shape‐memory alloy (SMA)–based soft actuators. The “smart braid” (Figure 2f) developed by Felt and Remy[[qv: 38a]] is another notable enabling sensing technology for soft actuators with self‐proprioception capabilities. However, they are yet to be exploited in other soft actuators other than McKibben muscles. Despite the rigid feature of magnets and Hall‐effect sensors, magnetic field–based curvature sensors[[qv: 42a]] can be a suitable solution for soft continuum robots, due to their high performance, low cost, small size, and easy integration. And, magnetic field–based tactile sensors^45^ have the potential to be implemented in soft robots as well. Sensors relying on elastomer‐based optical waveguides can also represent alternative solution as it was demonstrated in a soft prosthetic hand^27^ which will be discussed in detail in Section 2.2 as tactile sensing was implemented too.

### 2.2 Tactile Sensing

In the past decade, remarkable progress was attained in developing flexible thin electronic skins (tactile sensing skins),^46^ benefiting from advances in printing techniques, flexible (in)organic electronics, and advanced materials. Notable examples include an ultralightweight, tactile sensing array with integrated organic electronics,^47^ a fully printed flexible tactile skin for triaxis force and temperature sensing,^48^ etc. Detail descriptions and comprehensive discussions of electronics skins can be found in a few excellent review papers.^46^, ^49^ Several ultrathin, flexible electronic skins have been demonstrated in wearable and biomedical systems[[qv: 49a,50]]. A few flexible tactile sensors or sensing skins with integrated rigid electronics covered with soft protective layer have been integrated into conventional robotic systems (e.g., capacitive triangular skin on the iCub robot,^51^ and a hexagonal modular skin^52^). Technically, most of these skins are flexible, not highly stretchable, and their thin structures would be vulnerable for repeated physical contacts in robotics applications. Nevertheless, the mechanisms, materials, electronics, and fabrication technologies used in developing such thin skins can be explored and utilized for developing tactile sensing systems for soft robots.

**Figure 3:**
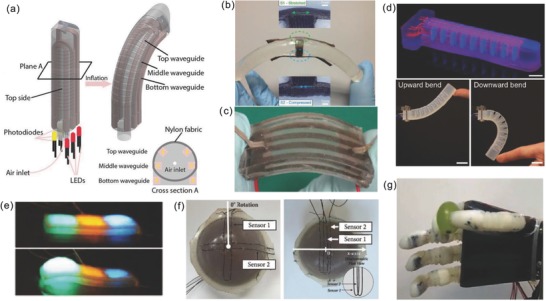
Examples of soft robotics and structures with tactile sensing. a) Soft robotic finger with integrated waveguide strain sensor for both proprioception and tactile sensing. Reproduced with permission.^27^ Copyright 2016, AAAS. b) A differential capacitive sensor pair that can distinguish bending curvature and external force. Reproduced under the terms of the CC‐BY 4.0 license.^54^ Copyright 2015, the Authors. Published by Macmillan Publishers. c) A hybrid sensor can detect strain and pressure simultaneously. Reproduced with permission.^55^ Copyright 2017, Mary Ann Liebert. d) A soft bending actuator with integrated curvature, inflation, and contact sensors via embedded 3D printing. Reproduced with permission.^56^ Copyright 2018, Wiley‐VCH. e) A soft crawling robot with tactile or deformation sensing capabilities. Reproduced with permission.^57^ Copyright 2016, AAAS. f) A universal granular jamming gripper with integrated strain sensors for object size recognition. Reproduced with permission.^58^ Copyright 2017, IEEE. g) A soft prosthetic hand with integrated capacitive sensors for bending and pressure measurement. Reproduced with permission.^59^ Copyright 2018, IOP Publishing.

Recently, some researchers have attempted to implement both proprioception and tactile sensing systems in soft robots. In 2016, Zhao et al.^27^ implemented stretchable optical waveguides as strain and pressure sensors in a pneumatic‐driven, soft prosthetic hand (**Figure** **3**a). This soft hand can detect the fingers' bending angle (proprioception) and feel the touch (contact force) at each fingertip (tactile sensing). Even though these sensors and wires are bulky and the tactile sensing ability is limited to single point pressure measurement at fingertips, it is demonstrated that the capabilities of the soft hand can be significantly enhanced by enabling mechanical perception. Earlier in 2013, the same transducer mechanism has been exploited for transparent tactile sensing skin.^53^ Notably, in 2015, Lucarotti et al.^54^ demonstrated that a sensing pair can closely follow the movement of a bending soft module, which can be used to discriminate bending curvature and contact pressure (Figure 3b). A differential configuration of two textile‐based capacitive sensors on a cylindrical elastomer was experimented. Totaro et al.^55^ from the same group developed a hybrid sensor that can simultaneously detect force and bending curvature of a soft structure by combining a piezoresistive strain sensor and an optical waveguide pressure sensor (Figure 3c). In this case, mechanics of the soft body was exploited to place the strain sensor in a way that can mechanically filter external pressure stimulus, ensuring that the strain sensor is only sensitive to bending. Very recently, Truby et al.^56^ reported a method for creating soft somatosensitive actuators via embedded 3D printing of ionogel‐based resistive sensors (Figure 3d). A soft robotic griper with proprioception and haptic feedback was developed by embedding curvature, inflation, and contact sensors through a multimaterial printing platform. This direct fabrication of fully integrated perceptive soft actuators would open enormous possibilities in the development of soft self‐sensing robots. Alternatively, Larson et al.^57^ developed a highly stretchable electroluminescent skin for a soft crawling robot that can either sense external pressures or the degree of deformation of the pneumatic chambers, during locomotion (Figure 3e), showing that skin deformation can also be communicated through luminescence. Here, soft hyperelastic capacitive sensors were built with ionic‐hydrogel electrodes and an elastomeric dielectric doped with Zn–phosphor powders.

In 2017, Hughes and Iida^58^ proposed a method to measure the strain location, amplitude, and direction on a soft deformable surface by using differential sensing pairs. The resulting sensorized vacuum jamming gripper is capable of detecting the size of objects (Figure 3f). In 2018, Rocha et al.^59^ integrated capacitive bending and pressure sensors on a soft prosthetic hand (soft skin and rigid skeleton), and a variety of grasping tasks were performed by this hand with sensorized fingers (Figure 3e). In 2018, Yang and Chen^60^ developed a soft finger with embedded pressure and position sensors using piezoresistive elastomer, for feedback control of a grasping task. These explorations are relatively preliminary, but they demonstrate that the capabilities of these sensorized soft robots are significantly enhanced.

To explicitly show the most common functionalities in soft robots and how these have been coped with sensing technologies for proprioception and tactile sensing, we summarize them in **Table** **1**. All these case studies are categorized according to the type and sensing functionalities of the robots, and sensing transducers and materials used are listed for each case.

**Table 1:** Summary of soft robotic case studies with sensing capabilities

Soft robots	Sensing functionality	Sensing transducers	Materials (electrodes)	Ref.
Soft pneumatic bending actuators	Curvature sensing	Resistive	Metal liquid	26, 30
		Resistive	Helical wire	61
		Resistive	Ionic liquid	43
		Piezoresistive	Nanocomposites	[[qv: 14b,31]]
		Piezoelectric	P(VDF/TrFE)a)	[[qv: 14a]]
		Optical	Engraved optical fiber	28
		Pneumatic sensor	Elastomer with enclosed air chamber	62
Soft omnidirectional actuators for MIS applications	Bending angle and direction	Resistive	Ionic liquid	33
		Resistive	Conductive yarn	34
		Optical	Optical fiber	35
McKibben muscles	Contraction length or circumference	Inductive	Smart braid	38, 63
		Resistive	Conductive yarn	37, 64
		Piezoresistive	Nanocomposites	36
Soft continuum robots	Shape reconstruction	Resistive	Textile	[[qv: 14c]]
		Resistive	Conductive yarn	34
		Magnetic	Hall sensors, magnets	42
		Optical (FBG)	FBG fibers	40, 65
		Piezoelectric	PVDF	41
		Inductive	Helical coils	66
Anthropomorphic hand	Curvature, elongation, and force	Optical	Stretchable waveguide	27
	Bending angle, contact force, proximity	Capacitive	Nanocomposites	59, 67
	Multidirectional bending angle, twist	Resistive	Liquid metal	[[qv: 22b]]
Crawling robots	Internal deformation or tactile sensing	Capacitive	Ionic hydrogel	57
Universal jamming gripper	Strain direction, location, and amplitude	Piezoresistive	Conductive thermoplastic elastomer	58

^a)^ poly(vinylidenefluoride‐*co*‐trifluoroethylene).

### 2.3 Sensor Configuration

**Figure 4:**
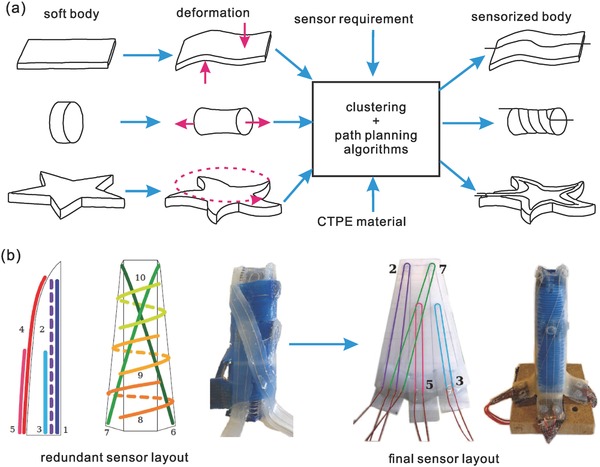
a) Sensing morphology: the strain vector aided sensorization method. Adapted under the terms of the CC‐BY license.[[qv: 22a]] Copyright 2014, the Authors. Published by MDPI. b) Step‐by‐step method to optimize the sensor configuration for designing a sensorized soft actuator. Adapted with permission.[[qv: 22b]] Copyright 2017, IEEE.

It is impossible to implement thousands of sensors in a soft robotic system like a biological system given that there are so many challenges in electronics, sensor size and space, wiring, powering, system integration, data communication and processing. Therefore, a smart sensor design and configuration strategy is needed to minimize the number of sensing nodes required to achieve a desired sensing capability, and to optimize the sensor configuration for the best performance. It is much more challenging to develop such kind of sensor configuration strategy for soft robots because of their largely deformable body and high number of DOFs. In 2014, Culha et al.[[qv: 22a]] developed a morphological sensing method called strain vector–aided sensorization of soft structures (**Figure** **4**a). In this method, deformations of the soft body are analyzed through modeling, and the optimal strain path that best characterizes a specific deformation of the soft structure can be found. Then, different motion patterns of a soft block can be discriminated by implementing strain sensing wires at the locations calculated through this method.^68^ In 2017, Wall et al.[[qv: 22b]] developed a step‐by‐step method for designing sensorized soft actuators. The number of sensors can be reduced by only implementing these sensors at the best location for detecting multiple deformations (Figure 4b). Both training and machine learning were involved to identify which sensors are the most effective and necessary for the required sensing functionality. The work is rather preliminary because of the large size and limited numbers of the sensors that probably affected the performance of the soft actuators, but it pointed out a clear direction and method to develop a soft sensing system wisely.

## 3 Sensing Technologies for Soft Robots

Driven by the demands of robotics and biomedical applications,[[qv: 46b,69]] a vast range of soft strain and tactile sensors have been developed, which provide enormous potential for implementing a compatible mechanical perception system for soft robots. Given that there are so many types of soft actuators and robots, it is impossible to design a universal sensing system. Generally speaking, to develop fully perceptive robots, sensors should meet the following basic requirements:

1) Be sufficiently compliant (low Young's modulus), not restricting or significantly changing the mechanical properties of the soft actuators;
2) Have limited dimensions and a spatial distribution such that free robotic movements are allowed;
3) Be resilient and durable to survive large strain (under over loading or driving) and thousands of deformation cycles without failure;
4) Endure interaction with outside world, e.g., show robustness to repeated mechanical stimulation in various environments from the most delicate (e.g., surgical robots) to the harsher ones (e.g., search and rescue robots);
5) The fabrication methods and the materials used should allow the sensing elements to be part of the soft body of the robots. Ideally, the sensing mechanisms should originate from the robot architecture, and/or can be implemented in the same materials as the soft body, or at least be such to limit stress concentration and adhesion issues.

**Figure 5:**
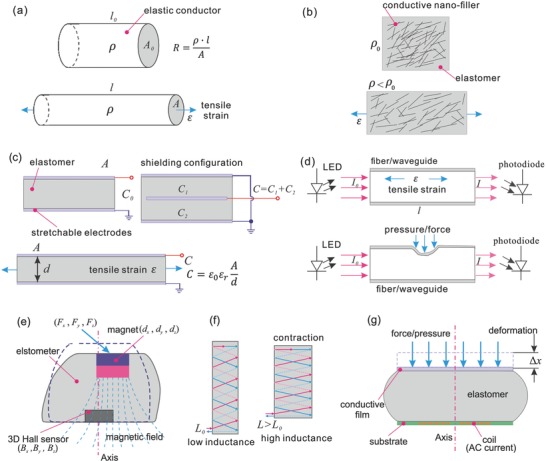
Transducer mechanisms for strain and pressure sensing: a) resistive sensor; b) piezoresistive sensor; c) capacitive sensor; d) optical fiber–/waveguide‐based pressure and strain sensor; e) magnetic tactile or deformation sensor; f) the “smart braid;” g) inductive tactile sensor.

There are several sensing technologies that hold promise for inventing new sensorized soft robots. In this section, we summarized these technologies which exploit different transducer mechanisms, spanning from resistive, capacitive, optical to magnetic and inductive (**Figure** **5**). We discuss and compare the advantages, limitations of these sensing technologies, and cases of implementing these sensors in soft robots are outlined. Besides these, there are a few other transducer mechanisms that have been used in soft robotics sensing, such as ultrasonic^70^ and pneumatic sensors.^62^ In the last decade, both triboelectric effect^71^ and piezoelectric effect^72^ have been exploited for self‐powering, flexible, 2D pressure mapping using novel materials and nanostructures. Details can be found in the review papers from Wang and co‐workers.^73^ Despite the appealing feature of self‐powering, they measure dynamic stimuli only, and the results are often affected by environmental conditions (e.g., humidity, temperature, air pressure, etc.). It would be challenging to develop a reliable soft sensing system based on these transducers for a deformable robot.

### 3.1 Resistive and Piezoresistive Sensors

Resistive and piezoresistive strain sensors^74^ measure the resistance variations caused by changes in geometry or in resistivity of conductive materials (Figure 5a). To achieve stretchable sensing form, resistive sensors exploit the flowable feature of conductive liquids^75^ embedded in elastomers or the stretchable feature of conductive polymers and hydrogels.^56^, ^76^ These conductive liquids include low‐melting point metal and metal alloys^77^ (e.g., mercury (Hg), eutectic gallium–indium alloy (EGaIn), gallium–indium–tin alloy (Galinstan)), and all kinds of ionic liquids (e.g., sodium chloride dissolved in water).^43^ Liquid metals have very good conductivity, but cannot work at temperatures lower than their melting point, and their density is generally much higher than most elastomeric substrates. Ionic liquids are light (low density), cheap, but have poor conductivity, and they often encounter large temperature drift due to the correlation between temperature and ion concentration, and poor long‐term stability due to electrolysis when an electrical current is applied. Despite the complexity of fabricating microchannels[[qv: 75a]] and the risk of leakage, conductive liquid provides enormous potentials to develop highly stretchable, high‐performance strain sensors.^78^ However, rigorous design is needed to ensure robust interconnections between solid wires and liquid channel.^79^ Liquid metal–based resistive strain sensors have been integrated into a variety of soft robots, like soft pneumatic bending actuators,^26^, ^30^ human fingers,^80^ and soft robotic hands.[[qv: 22b]] Ionic liquid–based resistive sensors have also been developed for bending angle and direction measurement of soft omnidirectional actuators,^33^ human activities' monitoring,[[qv: 78a]] and proprioceptive soft fluidic actuators.^43^ In these applications, conductive liquid resistive sensors were used as stretchable strain sensors to measure the bending curvature of soft actuators or angles of joints. Very recently, a self‐healable, highly stretchable (500%), conductive polymer composite has been synthesized and tested for strain and pressure sensing,^76^ which could be an interesting direction for developing stretchable resistive sensors. Conductive yarn has been also exploited for flexible and stretchable resistive sensors by using helical structures to make the conductive strands stretchable; they have been integrated into soft omnidirectional actuators,^34^ soft bending actuators,^61^ and McKibben muscles.^37^, ^64^

Piezoresistive sensors are based on elastomeric composites filled with conductive fillers (nanoparticles (NPs), wires, or flakes),^74^ both the resistivity and geometry are changed when strain or pressure is applied (Figure 5b). These nanocomposites have tunable mechanical and electrical properties and can be fabricated with simple processes. However, they often encounter large hysteresis and nonlinearity, slow responses, and long recovery time.^81^ Furthermore, there is a trade‐off between stretchability and sensitivity, as a high filler ratio is required to achieve high conductivity, but this results in an increased stiffening effect and lower stretchability.^82^ Recently, a new method has been applied for building resistive polydimethylsiloxane (PDMS) sensors^55^ based on the implantation of metallic NPs into elastomeric substrates by supersonic cluster beam implantation (SCBI). SCBI is known for not dramatically altering the mechanical properties of the polymeric substrates when significant metal volume concentrations are implemented,^83^ hence it could represent a good candidate for keeping the hyperelastic properties of the soft body materials. Conductive nanocomposite–based piezoresistive sensors have been well investigated for soft tactile sensors.^74^ They have been also integrated into a variety of soft actuators[[qv: 14b,31]] to measure bending curvatures. The piezoresistivity effect of conductive textile also has been utilized for soft tactile sensing^84^ and curvature sensing of soft continuum robots.[[qv: 14c]] A detailed analysis and discussion of the nanocomposite strain sensors mechanisms can be found in a review paper of Amjadi et al.^81^

In general, resistive and piezoresistive sensors require very simple readout electronics, are insensitive to electromagnetic interferences, and are flexible to be designed in different forms for various applications. The mechanical and electrical properties of these nanocomposites and conductive polymer materials can be tuned, but require expertise in material synthesis. Conductive liquid–based sensors are easy to use, but require complex manual procedures to fabricate and are difficult to miniaturize. Most resistive and piezoresistive sensors have poor sensitivity compared to other transducer mechanisms and they often encounter large hysteresis, nonlinearity, lower repeatability, and large temperature drift. In summary, they are relatively easy to be fabricated and integrated into soft robots, but have limited performance and bandwidth.

### 3.2 Capacitive Sensors

Capacitive sensors^85^ measure the capacitance variations caused by geometry changes when the elastic body is deformed (Figure 5c). The key toward stretchable sensing is to develop highly stretchable, conductive materials as electrodes. So far, conductive fabric,^85^, ^86^ nanocomposites,^87^ conductive polymer/hydrogel,^57^ bulking thin film,^88^ and conductive liquid^89^ have been deployed as stretchable electrodes. Capacitive sensors have good linearity, high sensitivity, large dynamic range, and rapid response. However, they are sensitive to environmental contaminants (e.g., oil, dust, liquid, vaporous water, etc.) and have proximity effect to conductive objects. To solve those issues, shielding techniques are needed (e.g., three‐electrode configuration), while they increase the fabrication complexity. Furthermore, stretchable capacitive sensors are sensitive to both pressure and strain, which are challenging to decouple.

Given the high performance and good linearity, capacitive tactile sensors have been well developed for soft tactile sensors, electronic skins, and soft strain sensors, even though there are not many application cases in soft robots yet, compared to resistive sensors. Recently, applications in wearables and soft robotic systems have started to grow rapidly because of the utilization of stretchable conductive textile as electrodes. Tactile strain sensors using textile electrodes have been engineered for detecting movement of human fingers and joints,^86^, ^90^ for making soft sensory sleeves for soft robots curvature estimation,^91^ for soft bending actuators,^29^ and for bioinspired soft robots.^54^, ^92^ Soft capacitive sensors using conductive nanocomposite electrodes have been used to curvature and touch sensing for a soft prosthetic hand.^59^, ^67^ Notably, a highly stretchable capacitive sensor using ionic‐hydrogel electrodes has been used in a soft pneumatic crawling robot for deformation or tactile sensing.^57^

### 3.3 Optical Sensors

Optical strain sensors detect the light variations (intensity, frequency, or phase) caused by strain or pressure applied to the light transmission medium (e.g., optical fiber,^28^ elastic waveguide^93^). The most common transduction mechanism is based on light intensity measurement, and it usually comprises a light source (i.e., a light‐emitting diode), a photodetector, and a light transmission medium (Figure 5d). Optical strain sensors are highly deformable, insensitive to any electromagnetic interference and environmental contaminants. They have been used for bending angle and direction monitoring of a soft omnidirectional actuator,^35^ closed loop control of a wearable pneumatic hand,^28^ and curvature and contact force sensing of a soft prosthetic hand.^27^ Optical fiber–based sensors have also been used for tactile sensing in a soft surgical manipulator,^94^ and prosthetic finger.^95^ Despite relatively complex electronics, the main advantage of optical sensors with respect to other technologies is to avoid electronic components and wires distributed on the sensing active area. Recently, an ultrathin, waveguide‐based optical system has been developed as a soft tactile sensor array.^93^ TacTip represents another type of optical sensors for soft tactile sensing by monitoring the deformation of a soft structure's skin through an embedded camera.^96^ A variety of TacTip sensors have been developed for different robotics and biomedical applications,^97^ but this solution still presents a bulky and rigid camera system that would be difficult to integrate in soft robots. Utilizing similar mechanism, Gelsight^98^ provides much better spatial resolution, but also relies on a camera system. FBG^99^ technology has also been introduced into soft strain sensing for soft continuum robots.[[qv: 40,65a]] Multiple FBGs can be fabricated on different longitudinal positions of one fiber, so strain and pressure distributions across the fiber length can be monitored by the electronics located at the ends of the fiber. FBGs show great potential to develop high performance, completely soft strain or pressure sensing array without any electronics located at the sensing site.^100^ The expensive and complex fabrication process, limited stretchability, and complex signal conditioning electronics are the main barriers for embedding FBG‐based sensors in soft robots.

### 3.4 Magnetic Sensors

Magnetic strain and tactile sensors comprise a magnetic source (e.g., permanent magnet), a magnetic field sensor (e.g., Hall‐effect sensor), and a soft medium,[[qv: 42b,45a]] as shown in Figure 5e. When the soft medium is stretched, compressed, or twisted, the magnetic field readout from the sensor varies because of the changes of the relative position and orientation of the Hall‐effect sensor, with respect to the permanent magnet. Using this concept, magnetic field sensors have been developed as tactile sensors for soft robotic hands[[qv: 45b]] and curvature sensors for proprioceptive soft robots.^42^ Magnetic sensors are compact, low in cost, deformable, highly sensitive, and easy for system integration. However, they are inherently vulnerable to external interferences coming from environmental magnetic field variations and their interaction with ferromagnetic objects.

### 3.5 Inductive Sensors

Inductive sensors measure the inductance variations caused by a few transducer mechanisms, e.g., coil geometry,^101^ mutual inductance,[[qv: 66a]] eddy‐current effect,^102^ and magnetic reluctance.^103^ By measuring the changes of inductance, deformation, strain, displacement, pressure, or dimensions of soft actuators and structures can be monitored (Figure 5f). In 2014, Felt and Remy[[qv: 38a]] developed an inductance‐based soft deformation and force sensor for McKibben muscles by forming stretchable helical coils on the fiber‐reinforced braid (“smart braid”). Changes in inductance and resistance of the helical coil represent the contraction length, and force, of the artificial muscle, respectively. Based on the principle of mutual inductance variations, the same group also developed an inductance‐based curvature sensor for continuum robots.^66^ Recently, Wang et al.^102^, ^104^ developed a new type of inductive tactile sensor based on eddy‐current effect, which is low in cost, of high performance, robust in harsh environments (e.g., underwater), and durable to repeated contact (demonstrated by the hammer strike test). New innovations and advancements are needed to overcome their complex signal conditioning electronics for widespread use.

### 3.6 Sensing Technologies for Soft Robots

In **Table** **2**, we summarize these sensing technologies that have been integrated in soft robots, actuators, and structures. These sensors are classified by the transducer mechanisms and the key materials used, sensing functions, and applications are also listed.

**Table 2:** Sensing technologies for soft robotics

Transducer mechanisms	Materials (electrodes)	Sensing functionality	Applications	Year	Ref.
Resistive sensors	Liquid metal	Bending curvature, force	Soft bending actuator	2016	26
		Strain or bending angle	Soft bending actuator	2016	30
		Strain and curvature	Soft robotics	2017	105
		Strain and curvature	Anthropomorphic hand	2011	80
		Multidirectional bending, twist	Soft robotic hand	2017	[[qv: 22b]]
	Ionic liquid	Bending angle and direction	Soft omnidirectional actuator	2015	33
		Contact force, bending angle	Robotic joint	2010	106
		Strain	Human movement monitoring	2017	[[qv: 78a]]
		Curvature or elongation	Soft bending and extending actuators	2017	43
	Conductive yarn	Length	McKibben muscle	2015	64
		Length	McKibben muscle	2016	37
		Bending angle and direction	Soft omnidirectional actuator	2015	34
	Helical wire	Curvature	Soft bending actuator	2016	61
	Ionogel (printed)	Curvature, inflation, contact force	Soft bending actuator	2018	56
Piezoresistive sensors	Nanocomposite	Curvature	Soft bending actuator	2008	[[qv: 14b]]
		Circumference	McKibben muscle	2009	36
		Curvature	Soft bending actuator	2017	[[qv: 31a]]
		Local strain	Soft bending actuator	2017	[[qv: 31b]]
		Bending, twisting, elongation	Soft structure	2014	[[qv: 22a,68]]
		Strain amplitude, location, direction	Universal jamming gripper	2017	58
		Pressure and curvature sensor	Soft robotic finger	2018	60
	Fabric	Contact force	Soft bending actuator	2016	84
		Pressure	Soft haptic device	2016	84
	Textile	Strain	Soft continuum robot	2012	[[qv: 14c]]
Capacitive sensors	Nanocomposite	Deformation, triaxis force	Pneumatic actuator	2016	107
		Curvature, proximity, pressure	Soft bionic hand	2017	59, 67
	Textile	Curvature	Sensory sleeve for soft robot	2017	91
		Curvature, contact force	Soft bending actuator	2017	29
		Multiaxis force	Bending actuator	2016	92
		Bending and contact force	Soft structure	2015	54
		Bending angle	Anthropomorphic hand	2017	86
		Pressure sensor	Anthropomorphic hand	2017	90
	Wrinkling film	Bending angle	Anthropomorphic arm	2017	[[qv: 88a]]
	Ionic hydrogel	Deformation/pressure	Soft crawling robot	2016	57
Magnetic	Hall sensors and permanent magnets	Curvature	Soft‐bodied robot	2015	42
		Tactile	Robotic fingertip	2017	[[qv: 45b]]
Inductive	Smart braid	Contraction length and force	McKibben muscle	2014, 2016, 2017	38, 63
	Coil	Bending angle and direction	Continuum robotic arm	2016, 2017	66
	Zigzag coil	Diameter	Urinary catheter (balloon)	2014	108
Optical	Optical fiber	Bending angle and direction	Soft omnidirectional actuator	2015	35
		Tactile	Soft omnidirectional actuator	2014	94
		Curvature	Wearable soft hand	2016	28
		Tactile	Prosthetic forefinger	2017	95
	Waveguide	Curvature, elongation, and force	Soft prosthetic hand	2016	27
	Contactless	Curvature	Soft‐bodied robot	2011	109
	FBG	Strain for shape reconstruction	Soft continuum robot	2014, 2016, 2018	40, 65
Piezoelectric	P(VDF/TrFE)	Curvature	Soft bending actuator	2007	[[qv: 14a]]
	PVDF	Shape reconstruction	Flexible beam	2014	41
Pneumatic	Air pressure sensor	Curvature or contact pressure	Soft bending actuator	2017	62
Ultrasonic	–	Length	Pneumatic actuator	2016	70
Hybrid	Optical waveguide/piezoresistive	Strain and pressure	Soft structure	2017	55

As it can be seen, many transducer mechanisms have been exploited as soft strain sensors or tactile sensors in soft robotics, but it is difficult to define the criteria to evaluate the performance of these sensors from the perspective of soft robotic applications. For example, for a soft strain sensor, the following criteria should be used to evaluate its performance: resolution (minimal detectable strain variation), stretchability (strain at break), dynamic range (ratio between effective sensing range to resolution), and response time. Then, there are also important factors to be considered in order to choose and design a strain sensing system for a soft robot, like: sensor size, robustness, durability, fabrication complexity, cost, power consumption, and the design flexibility and scalability. However, the latter are similar to the typical requirements of integrating components into conventional robots. There are some other aspects that should be considered for mechanical sensing in a soft robot. A major one is the different mechanical behavior that the same transducer may have when housed in soft bodied robots, which, in addition, may also change its stiffness when actuated (e.g., in pneumatic robots). Hence, the open question here is: what should be the adequate criteria to assess a sensing technology for a given soft robot? Since the distinction among actuation, sensing, mechanical body, and control is not clear in soft robots as in conventional ones, it remains an open issue for the scientific community to define the requirements that can weigh all aspects (the desired sensory feedback, robotic functionalities, actuation mechanisms, materials of the soft body, application scenarios, etc.).

## 4 Challenge

### 4.1 Multimodal Sensors

**Figure 6:**
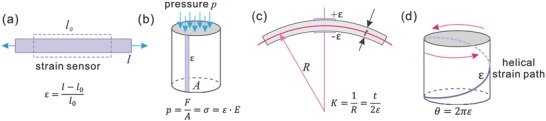
a) Definition of a stretchable strain sensor. b) A pressure sensor using a strain sensor. c) A bending curvature sensor using a pair of strain sensors. d) A twisting angle sensor using a stretchable strain sensor placed on helical path.

To achieve both proprioception and tactile sensing in a soft robot, an important issue regards the design and development of multimodal, stretchable sensors. They should be embodied into the soft actuator to measure strain, pressure, bending, and/or twisting in the soft body. As shown in **Figure** **6**, a stretchable strain sensor is a basic component that can be deployed in different designs to achieve all other sensing modalities. Therefore, pressure, bending, and twisting sensors can be designed using stretchable strain sensing wires, while they also can be directly formed by simple designs without using strain sensors at some cases, e.g., capacitive pressure sensor. Among all these sensing technologies listed in Section 3, (piezo)resistive sensors and capacitive sensors are more easily to be used to develop high‐density, ultrathin, pressure sensing arrays (electronic skins), while magnetic and inductive sensors have the advantages of being smoothly integrated into some robots for proprioception. Generally speaking, same transducer mechanisms should be deployed to develop sensors with different sensing modalities in a single robotic system to simplify fabrication, integration, signal conditioning, and control. While in some cases, the design and system integration can be much simpler by using different transducer mechanisms to form different sensing modalities with the expense of complex electronics. New innovations are needed to develop multimodal sensors that can meet the requirements of different soft robots. Ideally, a fully integrated device with sensing and actuating capabilities (e.g., proprioceptive soft actuators driven by conductive liquid^27^), and even computing at some extent, would significantly reduce the system complexity, boosting the development of perceptive soft robots.

### 4.2 Stretchable Conductive Materials and Structures

**Figure 7:**
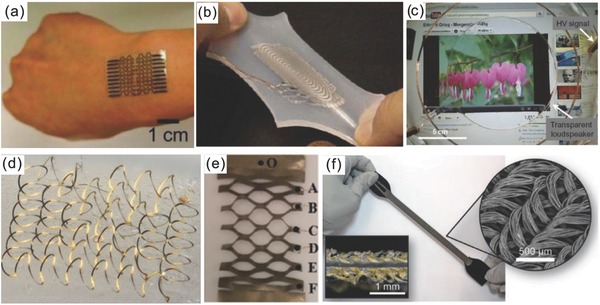
Examples of stretchable conductive materials and structures: a) Skin‐mountable strain gauges based on conductive nanocomposite. Reproduced with permission.^110^ Copyright 2012, Wiley‐VCH. b) Stretchable triaxis strain sensors based on liquid metal. Reproduced with permission.[[qv: 75a]] Copyright 2012, IEEE. c) Transparent loudspeaker using conductive ionic hydrogel. Reproduced with permission.^112^ Copyright 2013, AAAS. d) 3D buckling wire structures for soft electronics. Reproduced under the terms of the CC‐BY 4.0 license.^113^ Copyright 2017, the Authors. Published by Macmillan Publishers. e) A kirigami‐patterned stretchable film. Reproduced with permission.^114^ Copyright 2017, AIP Publishing. f) Stretchable capacitive strain sensors using textile electrodes. Reproduced with permission.^86^ Copyright 2017, Wiley‐VCH.

Conductive materials are the core of all sensing systems to form electrodes, wires, and interconnections of sensing devices and electronics. For instance, (piezo)resistive and capacitive sensors are the most prevalent strain and tactile sensors, they are well investigated and the electronic interface is simple and mature. Highly stretchable and conductive materials and structures are needed toward stretchable sensing devices. Electrodes and wires should have high stretchability (>200%) to achieve highly stretchable sensing devices, and have good conductivity (comparable to copper) to reduce power consumption and noise of the sensing electronics. For stretchable wires and interconnections, constant conductivity under varying strains is ideal to develop high‐performance and robust sensing systems. Unfortunately, most good conductors (metals and metal alloys) have a very poor stretchability (only 3–5%). Currently, there are two approaches to develop stretchable conductors (**Figure** **7**): material approach (e.g., nanocomposite,^110^ liquid conductor,^111^ conductive polymer/hydrogel,^57^, ^112^ etc.) and structural approach (e.g., bulking/wrinkling structure,^113^ kirigami patterning,^114^ textile,^86^ etc.). Despite the widespread use, nanocomposite, conductive polymer/hydrogel, and conductive textile have poor conductivity and there is always a trade‐off between conductivity and stretchability. Liquid metals are good conductors and highly stretchable, but complex fabrication and leakage risk limit their applications. Furthermore, the resistance of these materials varies with strain, which will change the characteristics of the sensing device and electronics. Recently, developments in buckling metal films or wires are promising because of the high stretchability, good conductivity, and negligible resistance change under strain, but it is very complex to fabricate these structures. Developments of printable stretchable interconnections were summarized in this review paper.^115^ Therefore, innovations in new stretchable conductive materials and simple fabrication and assembly techniques of stretchable metal structures are highly demanded.

### 4.3 Modeling Mechanical Sensing of a Soft Robot

Several computational techniques have been successfully used in the design of mechanical sensors for traditional robotics, such as microelectromechanical systems (MEMS)‐based sensors that exploit the advantages of semiconductor technologies. In this case, they can be modeled as single component with elastic behavior and small deformations, placed over a rigid substrate, and the mechanical coupling with the robot body is very limited. Oppositely, in soft robotics, the robot body can be as soft and deformable as the soft sensor itself, the mechanical coupling between the robot and the sensor will change the characteristics of an embedded soft sensor. For example, the skin of an air chamber in a pneumatic robot will be stiffer when it is inflated, which will change the characteristics of the strain and pressure sensor attached on it. It is challenging to do accurate modeling given the complex behavior and large deformation of these hyperelastic materials used. Moreover, in the case of interaction between a sensorized soft robot and an object, the contact behavior is difficult to predict through modeling as friction (that is, affected by the condition of the surfaces in contact) plays a key role.^116^ In this case, due to the material characteristics, the classical theory of Hertzian contact^117^ has several limitations, giving only approximate solutions, and complex numerical simulations are needed.

Taking into account all those aspects would make the modeling much more complex, involving advanced techniques with high computational costs, and the results might not match well with physical systems. Efforts are needed to develop more efficient and reliable techniques. Soft sensors are generally considered as single components, and the mechanical coupling with a soft substrate or with soft contacting objects has been overlooked. In the literature, soft sensor modeling is addressed by exploiting semianalytical^118^ or numerical^119^ approaches. Relevant and reliable results have been obtained both at the micro‐ and the macroscale, enabling the design of optimized structures.^120^

On the other hand, finite element methods (FEMs) have been developed to simulate soft robot movements and to implement real‐time control algorithms.^121^ Recently, Duriez and co‐workers provided an online open‐source platform, called SOFA,^122^ which is dedicated to medical simulations but can be used for soft robotic systems as well. In particular, FEM computes the nonlinear deformations of the robots at interactive rates (low frequency, order of few hertz). The model is completed by Lagrange multipliers at the actuation zones and at the end‐effector position. Then, an iterative algorithm uses a reduced compliance matrix to find the contribution of the actuators (position and/or force) that deform the structure, so that the terminal end of the robot reaches a given position. Rigid or deformable obstacles and the internal characteristics of the actuators can be integrated in the control algorithm as additional constraints. The two simulation loops (high and low frequency) are coupled in order to guarantee mechanical accuracy of the system over time.

In the previous examples, real‐time loops are implemented without the use of soft sensors. Integrating soft sensors is very beneficial, enabling more complex tasks and more accurate control of the soft robot. To simulate and optimize the soft sensors and actuators as a system, multiphysical phenomena (mechanics, actuation, and sensing transducers) with multiscale features would need to be modeled and simulated accurately. For such a complex model at system level, a trade‐off between computational cost and accuracy would need to be found, to provide a design strategy toward perceptive soft robots.

### 4.4 Electronic Interfaces

Electronics play a key role in any sensing systems, and ideally it should be integrated into the sensing devices and be distributed in large areas. Fully integrated electronic interfaces were implemented in some flexible electronic skins[[qv: 46b,47]], but they are not stretchable. Implementing large number of tactile sensors has been investigated in conventional robots, and the key design factors, challenges, and potential solutions have been discussed and analyzed in a review paper of Dahiya et al.^17^ Some of these aspects are in common for soft robots, but their highly deformable body makes it more difficult to implement electronic interfaces. In most soft sensing systems, electronic interfaces are placed on rigid bases far away from the sensing sites because conventional silicon‐based electronics are inherently incompatible with soft material–based systems. Substantial progress has been made in flexible organic electronics,^123^ but it is still far away to be massively utilized in sensing systems because of the limited performance, functionality, and stretchability. Very recently, the intrinsically stretchable transistor‐based skin electronics^124^ from Bao and co‐workers would be promising to develop truly stretchable electronics to be fully integrated into soft systems. Alternatively, bendable silicon electronics with some specific functions have been developed to achieve flexible electronics by reducing the thickness of the semiconductor circuit structure,^125^ but the stretchability is still very limited. Recently, island‐based stretchable electronic systems has been developed by utilizing high‐performance and mature silicon chips with stretchable interconnections,^113^, ^115^ while the fabrication and assembly require complex procedures and highly skilled manual operation. A platform that can repeatedly and accurately embed small‐size, bare silicon chips with stretchable interconnecting wires into elastic substrate would be a significant enhancement toward stretchable circuits for practical applications in soft systems.

Moreover, power transfer and data communication have been always an issue in a system with large number of distributed sensing nodes.^17^ Despite the development of some self‐powered electronic skins using triboelectric/piezoelectric generators or solar power,^126^ data communication still requires wires to connect all sensing nodes to the control board. It is very difficult to implement massive stretchable connecting wires in a soft system, particularly challenging when the sensing nodes are distributed on a large arbitrary surface or inside a 3D soft structure. Self‐contained sensing nodes would solve the wiring issue at the root, in which each sensing node can be wirelessly (/self‐)powered, and wirelessly communicated with the controller or neighboring nodes.^127^ This kind of sensing nodes can be truly distributed on any surface or inside a 3D soft body to form the mechanical perception system for soft robots.

### 4.5 Data Interpretation

Since soft robotics sensing is a rather new field, there are not many studies on data interpretation of the sensing systems yet. Despite the developments in soft robotics proprioception, the core science has not been investigated; most of existing developments only involve a single sensor for bending angle measurement. Current shape reconstruction algorithms for soft continuum robots are oversimplified, and they do not address more complex investigations, e.g., on twisting or local deformation of the soft body. Moreover, accurate shape reconstruction methods for arbitrary shapes of soft robots have not been investigated. Therefore, advanced data processing algorithms that can utilize the raw data from all sensors to interpret them as meaningful mechanical perception information (shape, deformation, contact, pressure, shear force, etc.) are needed for soft robots. For example, algorithms transferred from electrical impedance tomography are used to interpret the electrical current signals to multipoint, multidirectional strain mapping of a 3D soft structure made of piezoresistive nanocomposites.^128^ In the case of a soft robotic fingertip with randomly distributed receptors, machine learning was employed to process the signals for discriminating several materials.^21^ Therefore, strategies and algorithms are needed to move forward on developing new tools and frameworks to interpret raw data from sensors to perception of information. In the meantime, system level modeling, data training, and machining learning^129^ can be employed to significantly improve the accuracy and efficiency of data interpretation.

### 5 Conclusion

In this work, we focus on the detection of mechanical cues in soft robots for both proprioception and tactile sensing. We emphasize that this is the foundation for intelligent soft robots, which relies on developing reliable and accurate sensing technologies. Our aim is to provide a set of coordinates for researchers in soft robotics sensing, to establish new directions toward fully integrated, mechanically perceptive soft robots. In this regard, we highlight the general requirements for sensors that can be seamlessly integrated into soft robots. Developments in all aspects of soft robotics sensing (proprioception, tactile sensing, sensing morphology, and sensor configuration) are summarized and discussed. The sensing technologies that have been and can be literally integrated into soft robots are summarized, and their pros and cons are discussed. Despite all these achievements, soft robotic sensing is still at its infancy, and there are many challenges to overcome toward autonomous soft robots. Innovations in robust and high‐performance multimodal sensors, stretchable conductors for electrodes and interconnections, fully integrated and/or wireless electronic interfaces, modeling and data interpretation methods are highly demanded. We argue that while basic criteria, including but not limited to resolution, dynamic range, stretchability, and response time, should be used to evaluate the performance of a sensing component, they are not adequate to assess its sensing capabilities in soft robots. Hence, a main challenge for the scientific community regards defining new criteria that consider the sensors as part of the soft robotic systems instead of discrete components. The sensory responses should be evaluated for a range of robotic tasks in function of the actuation mechanism, and also depending on the scenario in which the robot is moving. The definition of these criteria could represent the means through which several scientific communities involved (e.g., material science, engineering, biology) can communicate and collaborate, inspiring new ideas for innovative solutions toward perceptive soft robots.

## Conflict of Interest

The authors declare no conflict of interest.
